# Potential Hepatoprotective Activity of Super Critical Carbon Dioxide Olive Leaf Extracts against CCl_4_-Induced Liver Damage

**DOI:** 10.3390/foods9060804

**Published:** 2020-06-18

**Authors:** Amani Taamalli, Anouar Feriani, Jesús Lozano-Sanchez, Lakhdar Ghazouani, Afoua El Mufti, Mohamed Salah Allagui, Antonio Segura-Carretero, Ridha Mhamdi, David Arráez-Roman

**Affiliations:** 1 Department of Chemistry, College of Sciences, University of Hafr Al Batin, P.O. Box 1803, Hafr Al Batin 39524, Saudi Arabia; ataamalli@uhb.edu.sa; 2 Laboratoire de Biotechnologie de l’Olivier, Centre de Biotechnologie de Borj-Cedria, B.P.901, Hammam-Lif 2050, Tunisia; ridha.mhamdi@cbbc.rnrt.tn; 3 Research Unit of Macromolecular Biochemistry and Genetics, Faculty of Sciences of Gafsa, Gafsa 2112, Tunisia; ferianianwer@yahoo.fr (A.F.); ghazouani2005@yahoo.fr (L.G.); mufti.afoua-90@hotmail.com (A.E.M.); 4 Research and Development Functional Food Centre (CIDAF), Health Science Technological Park, Avenida del Conocimiento 37, Edificio BioRegión, 18016 Granada, Spain; ansegura@ugr.es (A.S.-C.); darraez@ugr.es (D.A.-R.); 5 Department of Analytical Chemistry, Faculty of Sciences, University of Granada, 18071 Granada, Spain; 6 Laboratory of Animal Ecophysiology, Faculty of Sciences of Sfax, Sfax 3018, Tunisia; amsallagui@yahoo.fr

**Keywords:** olive leaves, supercritical fluid extraction, antioxidants

## Abstract

Virgin olive oil has demonstrated its effective activity against oxidative stress. However, data on the bioactive effect of olive leaves or their major constituents on the liver are scarce. The present research work was conducted to evaluate the hepatoprotective effects of supercritical carbon dioxide (SC-CO_2_) extracts from fresh and dried olive leaves on hepatotoxicity caused by carbon tetrachloride (CCl_4_) in rat models. For this purpose, healthy albino rats of 180–250 g weight were used. The assessment of biochemical markers was carried out on blood and liver tissue. Then, a histopathological study was carried out on liver tissue. The obtained results showed that fresh and dried olive leaf extracts ameliorate the perturbed biochemical parameters caused by CCl_4_ treatment. Furthermore, the results registered for the histopathological study are in accordance with the biochemical parameters and the protective capacity of SC-CO_2_ extracts against DNA damage, indicating that olive leaf extracts helped to improve liver fibrosis caused by CCl_4_ treatment.

## 1. Introduction

Oxidative stress constitutes a disturbance characterized by an imbalance between the generation of free radicals and antioxidant defenses [1]. The reactive oxygen (ROS e.g., O2^−^, OH, ROO) and nitrogen species (RNS, e.g., NO, ONOO^−^) are generated in a variety of intracellular processes and their overproduction produces cell damage in lipids, proteins and DNA [2].

The liver is the largest organ in the vertebrate body and the site for intense metabolism [3]. It is involved in several vital functions and it has a great capacity to detoxify toxic substances and synthesize useful principles. Therefore damage to the liver inflicted by hepatotoxic substances is of grave consequences [4]. The hepatotoxin CCl_4_ has been used as a model component causing cellular necrosis in the liver. It induces hepatotoxicity in rats, rabbits and humans [5] and mediates its hepatotoxicity after biotransformation by hepatic microsomal cytochrome 450 (CYT 450) to generate trichloromethyl free radicals which launch attacks on membrane proteins, thiols and lipids. This leads to the peroxidation of the lipid membrane which results in necrotic cell death [6]. To prevent such disease, both enzymatic and non-enzymatic mechanisms are present in the cell [7]. The body protects itself from oxygen free radical toxicity by enzymatic antioxidant mechanisms (e.g., glutathione peroxidase, GSH; glutathione reductase, GR; superoxide dismutase, SOD; and catalase, CAT) and by non-enzymatic antioxidants (e.g., vitamins, uric acid, albumin, bilirubin, and many others) [8].

Liver pathologies constitute a serious global health issue despite recent therapeutic advances. Nevertheless, some plants have been used for liver disorders and showed to be therapeutically useful agents [5]. Diverse plant extracts were assessed for their hepatoprotective effect against different experimentally induced liver toxicities [9,10,11,12,13,14,15].

From the large diversity of the plant components, special importance has been assigned to their polyphenols, which have shown their capacity to counteract oxidative stress through various mechanisms [16]. An interesting approach used to obtain such compounds is to source them from food industry residues, which are in general disposed of or used to produce animal feed [17]. The olive tree (*Olea europaea*, L.), amongst the oldest known cultivated trees in the world and mentioned in the Holy Qur’ân and Ahadith [18], has been known for its long history of medicinal and nutritional values. Historically, olive leaves were used as a folk remedy for combating fevers and other diseases, such as malaria. Numerous researchers showed the important role of this plant in improving cardiovascular risk factors [19], cancer [20] and other diseases. A recent publication reviewed the relevant role of phenolic compounds present in *Olea europaea*, L. products and by-products in human health [21], while another review focused on the potential protective effect of secoiridoids from *Olea europaea* L. in cancer, cardiovascular, neurodegenerative, aging-related, and immune-inflammatory diseases [20].

Olive leaves constitute a huge abundant residue resulting from olive tree pruning and olive oil processing. It has been estimated that olive pruning produces 25 kg of olive leaves and twigs per tree annually. In addition these by-products present, only about 10% of olives actually arrive at the mills [22]. All of this makes olive leaves a very interesting, cheap source with potentially useful bioactive components such as secoiridoids, triterpenes, lignans, and flavonoids [23]. Oleuropein and hydroxytyrosol, as major compounds of olive leaves, have been reported to exert numerous pharmacological properties, including anticancer, antidiabetic, and anti-inflammatory activities [14]. Recently, the hepatoprotective activity of olive leaves against damage induced by CCl_4_ [24,25], cadmium [26,27], paracetamol [28], thioacetamide [29], ethanol [5], fluoxetine [30] and deltamethrin [31] has receieved increasing interest in animal experiemts. However, available data about the hepatoprotective effect of olive leaf extracts or their major constituents are still scarce. Hence, there is not enough literature about the hepatoprotective effect of olive leaves against CCl_4-_induced damage. The existing published articles focused on methanolic or butanolic extracts of dried olive leaves obtained by Soxhlet apparatus [24] and ethanolic extract from dried olive leaves standardized to 16–24% of oleuropein [25]. As far we know, this is the first study on the hepatoprotective effect of Tunisian olive leaves on CCl_4_ -induced toxicity. 

Nowadays, supercritical fluid extraction (SFE), an environmentally friendly and selective technique, has become one of the most popular green extraction techniques [32] used mainly in large-scale industrial applications. This technique has several advantages such as high efficacy, rapidity, non-toxicity, the absence of thermal degradation resulting a very high quality extracts, and the possible direct coupling to analytical instrumental technique [33] with a particular green interest due to the use of supercritical fluids such as CO_2_ instead organic solvents [34]. It has been increasingly used in recent years around the world for the processing of nutraceuticals as a ‘‘natural’’ alternative to traditional solvent-extraction processes [35]. It has acquired some relevance for the extraction of polyphenols from plant sources [36]. No previous literature reported the evaluation of olive leaf supercritical fluid extract for its hepatoprotective activity. In our present work, we used fresh and dried leaves from which extracts were obtained using a green advanced extraction technology. 

Taking into account all these aspects, this study aimed to assess and compare the hepatoprotective capacity of supercritical carbon dioxide (SC-CO_2_) extracts of fresh and dried olive leaves in CCl_4_-induced toxicity in rat models. For this purpose, serum biochemical parameters alanine aminotransferase (ALT), aspartate aminotransferase (AST), alcaline phosphatase (ALP and lactate dehydrogenase (LDH)), liver tissue parameters malondialdehyde (MDA), protein carbonyls (PC), total superoxide dismutase (SOD), catalase (CAT) and glutathione peroxydase (GPX) in addition to histopathological and DNA damage assays were evaluated. As far as we know, this is the first evaluation of the hepatoprotective activity of (SC-CO_2_) extracts from fresh and dried olive leaves acquired via green technology. Moreover, no available literature evaluated the effect of olive leaf extracts containing phenolic compounds and triterpenoid on induced hepatotoxicity in rats.

## 2. Materials and Methods 

### 2.1. Plant Material, Supercritical CO_2_ Extraction and Chemical Characterization

Olive leaves from the El Hor olive variety cultivated in Kairouan (center of Tunisia) were collected in January 2018 (voucher code OE00182). The cultivar was identified by Pr. Mokhtar Zarrouk. Dried leaves were kept under shade at 25 °C and fresh leaves were ground using an Ultra Centrifugal Mill ZM 200 (RetschGmbh, Haan, Germany). Extraction of phytochemicals was carried out according to a previously reported method [37]. Briefly, 10 g of ground leaves were homogenized with sea sand in a ratio 1:1.5 (m/m), loaded onto the extraction vessel, packed with glass wool and pressurized until working conditions. Extractions were carried out at 150 bar and 40 °C using a mixture of CO_2_ and ethanol (6.6%) as the extraction solvent which passed through the extraction cell for one hour at 23 g/min. The obtained extract was finally collected and the solvent was evaporated under vacuum at 38 °C. The extraction yields were 9.3% and 16.7% for fresh and dried leaves, respectively.

Olive leaf extracts were analyzed for their phenolic and triterpenoids composition by high performance liquid chromatography coupled to electrospray ionization and time of flight mass spectrometry (HPLC-ESI-TOF/MS). To achieve this goal, SFE extracts were reconstituted in an extraction co-solvent up to a concentration of 5000 mg/L and filtered through a 0.2 μm polytetrafluoroethylene (PTFE) syringe filter prior to further analysis. Qualitative and quantitative determination of both phenolic compounds and terpenoids were carried out according to the previously reported method [38]. Quantitation was carried out using available commercial standards of six phenolics and two terpenoids: hydroxytyrosol, vanillin, luteolin, ferulic acid, (+)-pinoresinol, oleuropein, oleanolic, and maslinic acids. These standards were purchased from Sigma–Aldrich (St. Louis, MO, USA), Arbo Nova (Turku, Finland), and Extrasynthèse (Lyon, France). The stock solutions containing these analytes were prepared by dissolving the appropriate amount in methanol or methanol/water (50/50, *v*/*v*) at a concentration level of 1 mg/L. All calibration curves showed good linearity over the range of study with a minimum value of R^2^ = 0.994. 

### 2.2. Experimental Animals and Toxicity Test

Healthy adult male albino rats weighing 180–250 g were used. The animals were provided from Pasteur institute and housed in a plastic cage under controlled temperature (24 ± 2 °C), 12 h light/dark cycle and 55 ± 5% relative humidity. The animals were guarded in the laboratory (Faculty of Sciences, Gafsa, Tunisia) for seven days before experiences with free access to standard laboratory food (SNA –Sfax) and water ad libitum. Animals were treated in accordance with guidelines and according to the Medical Ethics Committee for the Care and Use of Laboratory Animals of the Pasteur Institute of Tunis, Tunisia (approval number: FST/LNFP/Pro 152012). 

The toxicity of SC-CO_2_ extracts of fresh leaves and dried leaves mixed with corn oil was tested by gavage administration of four doses at 5, 10, 20 and 30 mg/kg body weight (b.w.). Animals (*n* = 6 in each group) were carefully controlled for progress of any toxicological symptoms at 30 min and then 4, 12, 24 and 48 h. 

### 2.3. Animals and Experimental Design of the CCl_4_-Induced Hepatotoxicity

**Scheme 1:**
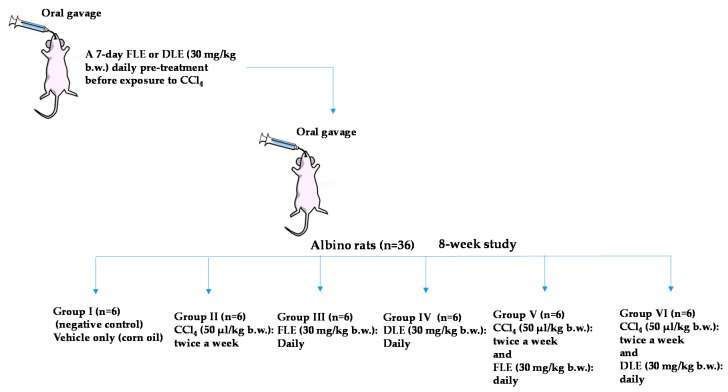
Graphical scheme summarizing the experimental design, *n*: number of rats.

To test the potential hepatoprotective effects of olive leaf extracts, experiments were conducted in six groups according to the following administration (Scheme 1): group I—negative control with administration of vehicle only (corn oil); group II—administration of carbon tetrachloride (CCl_4_) in corn oil 50 µL/kg [39]; group III—daily administration of fresh leaf extracts dissolved in corn oil (FLE, 30 mg/kg); group IV—daily administration of dried leaf extracts dissolved in corn oil (DLE, 30 mg/kg); group V—administration of FLE (30 mg/kg) and simultaneous treatment with CCl_4_ (50 µL/kg) +; group VI—administration of DLE (30 mg/kg) and simultaneous treatment with CCl_4_ (50 µL/kg). 

The CCl_4_ was administered by gastric gavage twice per week (on every Tuesday and Thursday) over eight weeks. A daily pretreatment with FLE or DLE was realized 7 days before CCl_4_ exposure and then daily throughout the research work by gastric gavage. The rats were sacrificed at the end of eight weeks and 24 h after the last dose of the hepatotoxin.

### 2.4. Analysis of Liver-Damage Serum Markers 

Blood samples were obtained by cardiac puncture. After coagulation, the samples were centrifuged at 3000× *g* for 10 min and serum was stored at −20 °C for further analysis. Levels of alanine aminotransferase, aspartate aminotransferase, alcaline phosphatase and lactate dehydrogenase were determined by spectrophotometric assays according to the commercially available diagnostic kits (Biomaghreb, Ariana, Tunisia).

### 2.5. Biological Determination of Liver Tissue

#### 2.5.1. Preparation of Tissue Extracts

The liver tissues were homogenized into 2 mL of ice-cold lyses buffer (pH = 7.4) using a grinder (homogenizer ultra-turrax). Then, the homogenate was centrifuged (12,000 rpm, 4 °C) for 15 min. The obtained supernatant was frozen at −20 °C to determine the oxidative stress biomarkers: malondialdehyde (MDA), protein carbonyls (PC), SOD, CAT and GPX.

#### 2.5.2. Measurement of Lipid Peroxidation and Protein Oxidation in Hepatic Tissue

The lipid peroxidation was estimated in control and treated rats by the quantification of thiobarbituric acid-reactive substances (TBARS) using the method described previously [24]. Briefly, the solution containing 100 µL of liver tissue extract and 100 µL of trichloroacetic acid (TCA, 5%) was centrifuged at 4000× *g* for 10 min. Then, in a boiling water bath, 100 µL of the supernatant was incubated with 200 µL of thiobarbituric acid reagent (TBA, 0.67%) for 15 min. The absorbance was read at 530 nm, the degree of lipid peroxidation was measured as thiobarbituric acid reactive substances (TBARS) and the result was expressed as nmol of MDA equivalents per gram of tissue (nmol MDA equivalents/g tissue). 

The protein carbonyl (PC) level was determined using the method described in the literature [40]. In this method, the carbonyl group of proteins was measured in the resulting pellets by reaction with 2,4-dinitrophenylhydrazine to form protein hydrazone which was measured spectrophotometrically at 370 nm.

#### 2.5.3. Determination of Enzymatic Antioxidants Activities

Total SOD activity, expressed as units per milligram of protein, was determined by measuring the inhibition of pyrogallol activity [41]. One unit (U) corresponded to the enzyme activity necessary to inhibit the oxidation of half of the pyrogallol. 

Catalase activity was determined in tissue supernatants using hydrogen peroxide (H_2_O_2_) as substrate according to the literature [42]. H_2_O_2_ degradation was accompanied by a decrease in absorbance and it was confirmed by measuring the absorbance at 240 nm for 1 min, and the enzyme activity was expressed as mmol H_2_O_2_ consumed per minute per milligram of protein.

Glutathione peroxidase activity was assayed by the subsequent oxidation of NADPH at 340 nm, using the method described by Flohé and Günzler [43], and the results were expressed as nmol of GSH oxidized per minute per milligram of protein.

Finally, protein concentration in liver homogenates was measured by the Bradford technique ((BCA) Protein Assay Kit, Pierce Biotechnology Inc., Rockford, USA) with bovine serum albumin (BSA) as standard.

### 2.6. Histopathological Study

The liver samples of the rats were fixed in 10% buffered formalin. After fixation, the tissues were dehydrated in a graded series of alcohol, cleared in xylene, embedded in paraffin, and cut into 5 µm thick slices. These serial tissue sections were stained with hematoxylin and eosin (HE) for routine histological examination. Sirius Red and Masson’s trichrome staining were used to visualize the collagen deposit in hepatic tissue, which was coloured blue using Masson’s trichrome and red by Sirius Red staining. The specimens were observed and photographed through a light microscope (Olympus CX31, Hamburg, Germany).

### 2.7. DNA Fragmentation Assay

Genomic DNA in the liver tissue of control and treated rats was isolated by phenol–chloroform DNA extraction method. The separation of intact and fragmented DNA fractions was visualized in an agarose gel by ethidium bromide staining, following the protocol described in the literature [44].

### 2.8. Statistical Analysis 

Statistical analysis was performed using GraphPad Prism 4.02 (GraphPad Software, San Diego, CA, USA). One-way analysis of variance (ANOVA) together with Tukey test were applied to observe significant differences between the tested treatments at a significance level (*p* < 0.05).

## 3. Results

### 3.1. Identification of Phenolic and Triterpenoid Composition in SC-CO_2_ Olive Leaf Extracts

In Table 1 are summarized the different compounds detected in both FLE and DLE. The analysis of the extract composition permitted the chemical characterization of 16 compounds. Quantitative results showed in this table include the amount of each identified compound in the administered dose to evaluate the hepatoprotective effects. 

A clear qualitative and quantitative difference was observed between fresh and dried leaves. In fresh leaves only vanillin, lignans (pinoresinol and acetoxypinoresinol), diosmetin and triterpenoids (maslinic, ursolic and oleanolic acids) were detected. This could be related to the impact of drying on the quality and the composition of olive leaf extract. Regarding the administered dose, acetoxypinoresinol was the major phenolic compound and constituted 37 µg in FLE, whereas in DLE, oleuropein formed the major phenolic compound (42 µg) followed by hydroxytyrosol (19 µg). Regarding triterpenoids, ursolic acid was the major compound in both extracts, being higher in fresh olive leaf extract.

### 3.2. Biochemical Measurements

#### 3.2.1. Effect on Plasma Biochemical Markers

Table 2 summarizes the impact of the hepatotoxin and olive leaf extract administration on liver function tests. Compared to the control group, the ALT, AST, ALP and LDH activities in serum of rats treated with CCl_4_ increased significantly (*p* < 0.001) after eight weeks reaching 336, 417, 231 and 261 U/L, respectively, indicating acute hepatocellular damage (Table 2). Nevertheless, the oral administration of olive leaf extracts (dose of 30 mg/kg b.w.) during intoxication showed a significant decrease in AST, ALT, ALP and LDH activities compared to CCl_4_ only-treated rats. 

The observed decrease was more pronounced in terms of AST activity when DLE was administered (389 U/L, *p* < 0.001) during CCl_4_ toxicity. Contrastingly, FLE administration in intoxicated rats caused a slightly more important decrease than with DLE administration in terms of ALT, ALP and LDH activities (Table 1). 

#### 3.2.2. Lipid Peroxidation and Protein Carbonyls

The effect on lipid peroxidation and protein carbonyls contents are summarized in Table 3. For CCl_4_-intoxicated rats, the TBARS level increased significantly (4.6 nmol MDA equivalents/g tissue) when compared with the control group (1.13 nmol MDA equivalents/g of tissue). Nevertheless, treatment with olive leaf extracts significantly reduced the lipid peroxidation level in CCl_4_-treated rats to 2.9 nmol MDA equivalents/g tissue in the case of FLE + CCl_4_ and 2.7 nmol MDA equivalents/g of tissue in the case of DLE + CCl_4_. 

Likewise, protein carbonyl amounts significantly increased (*** *p* < 0.001) further CCl_4_ intoxication (2.7 nmol/mg protein) in comparison to the control group (1 nmol/mg protein). However, FLE pretreatment of intoxicated rats showed a more pronounced effect than DLE pretreatment when compared to intoxicated rats (1.6 nmol/mg protein) with values of 1.0, 2.7 and 2.03 nmol/mg protein for control, CCl_4_ and DLE+ CCl_4_ treatment, respectively. 

A significant increase (** *p* < 0.01) was registered in terms of SOD, CAT and GPx activities among those in the CCl_4_-treated group (141 U/mg protein, 136 µmol of H_2_O_2_ destroyed/min per mg protein and 100 nmol of NADPH oxidized/min per mg protein, respectively) when compared to the control group (89 U/mg protein, 109 µmol of H_2_O_2_ destroyed/min per mg protein and 83 nmol of NADPH oxidized/min per mg protein, respectively). In contrast, SOD, CAT and GPx activities significantly decreased (*p* < 0.05) in intoxicated rats pretreated with DLE or FLE when compared to the rats who received only CCl_4_ (Table 3).

#### 3.2.3. Evaluation of Enzymatic Antioxidant Activities

Table 3 also shows the effects of olive leaf extracts on the antioxidant enzymes. It is noteworthy to say that CCl_4_ caused a significant increase in SOD, CAT and GPx activities (141 U/mg protein, 136 µmol of H_2_O_2_ destroyed/min per mg protein and 100 nmol of NADPH oxidized/min per mg protein, for SOD, CAT and GPx, respectively) when compared to the control group (89 U/mg protein, 109 U/mg protein and 83 U/mg protein, for SOD, CAT and GPx, respectively). Contrastingly, olive leaf extract administration caused a significant decrease in the activity of such enzymes when compared to CCl_4_-treated animals, resulting in levels close to those of the control group, especially for GPx activity (81 and 87 nmol of NADPH oxidized/min per mg protein, respectively, in comparison to the control rats with 83 nmol of NADPH oxidized/min per mg protein). 

### 3.3. Histopathological Examinations

#### 3.3.1. Hematoxylin and Eosin (HE) Staining 

**Figure 1:**
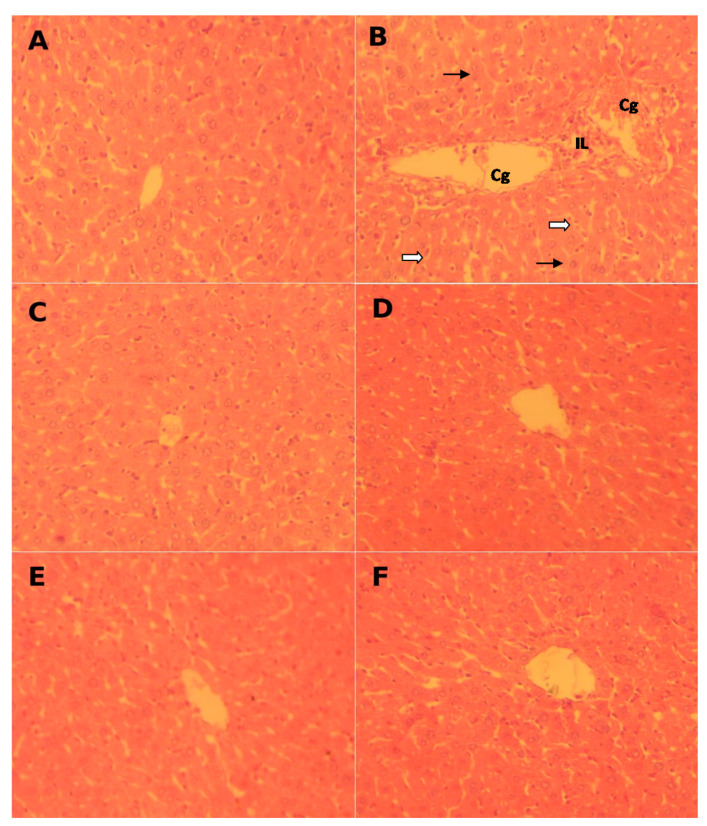
Pathological histology analysis in rat liver tissues after HE staining (magnification: 200×): (**A**) controls, (**B**) CCl_4_-treated (CCl_4_); (**C**) FLE-treated (FLE), and (**D**) CCl_4_-treated co-treated with FLE (CCl_4_ + FLE). (**E**) DLE-treated (DLE), and (**F**) CCl_4_-treated cotreated with DLE (CCl_4_ + DLE). Each microphotograph shows a section of an individual liver. Extensive focal necrosis (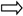), a moderate Kupffer cell hyperplasia (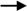), congestion and dilatation of the central vein (Cg) and leukocyte infiltration (LI).

(HE) staining of hepatic tissues of control and all treated rats are demonstrated in Figure 1. As it can be observed, the figure shows the hepatoprotective effect of FLE and DLE after CCl_4_ hepatotoxin administration.

The control rats’ hepatic sections revealed a normal form of hepatic cells; polyhedral hepatocytes and sinusoids boundaries exhibited a single layer of fenestrated endothelial and Kupffer cells (Figure 1A). FLE and DLE alone had no significant effects on liver histology (Figure 1C,E) whilst hepatoxin administration resulted in cells injury (Figure 1B). This was marked by extensive focal necrosis, a moderate Kupffer cell hyperplasia, congestion and dilatation of the central vein, and leukocyte infiltration. Finally, following the administration of CCl_4_ + FLE or CCl_4_ + DLE, focal neutrophils were infiltrated in some space and a number of centrilobular veins were congested (Figure 1D,F).

#### 3.3.2. Sirius Red and Masson’s Trichrome Staining

**Figure 2:**
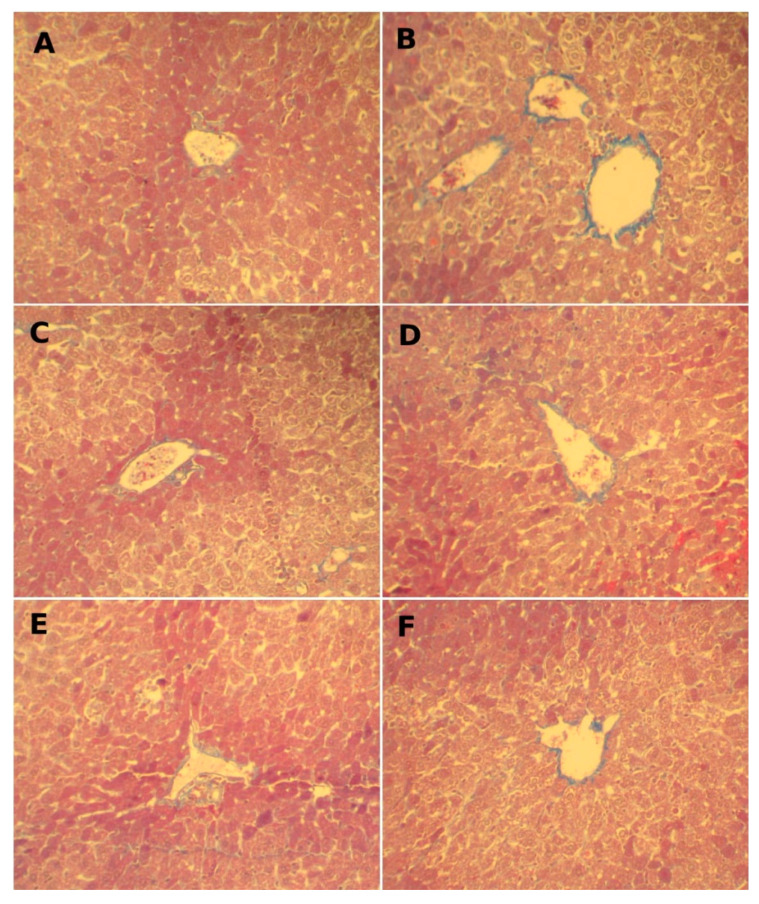
Effect of FLE and DLE on hepatic connective tissue fibrosis caused by CCl_4_ treatment: assessment by Masson’s trichrome stain (G × 200). (**A**) Control, (**B**) CCl_4_-treated, blue-stained zone indicates increasing fibrosis. (**C**) FLE-treated (30 mg/kg), and (**D**) co-treated with CCl_4_ and FLE (30 mg/kg); (**E**) DLE-treated (30 mg/kg) showing normal structure and (**F**) co-treated with CCl_4_ and DLE (30 mg/kg) showing results close to the control.

**Figure 3:**
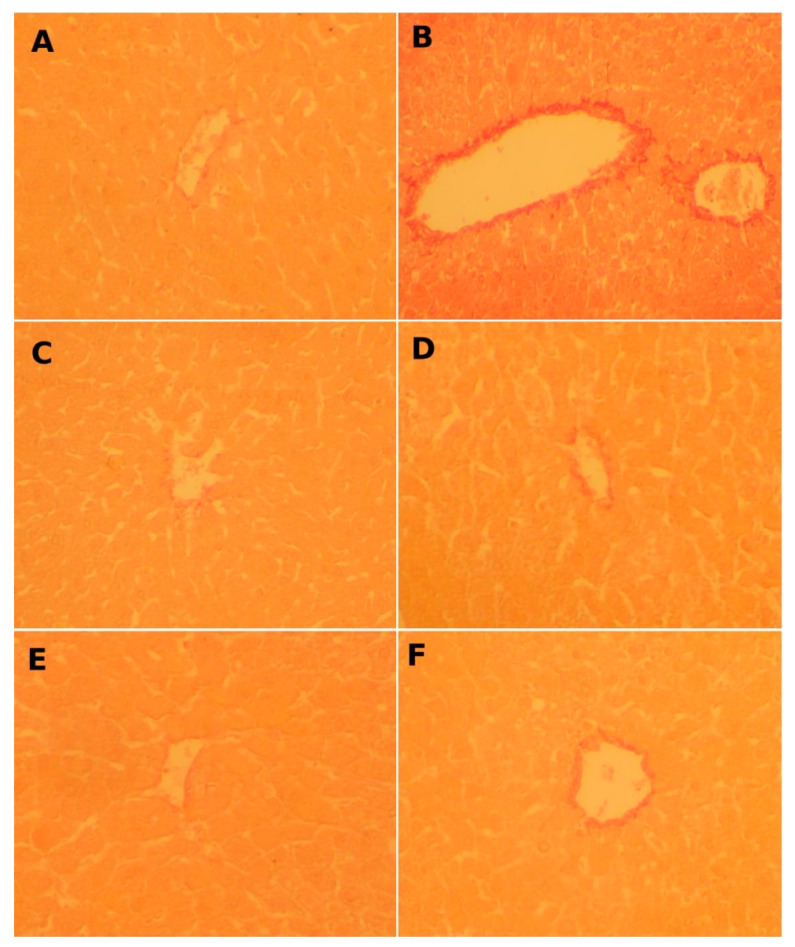
Micrograph showing the effect of FLE and DLE on hepatic fibrosis caused by CCl_4_. Assessment by Sirius red stain (G × 200). (**A**) Control, (**B**) CCl_4_-treated, showing intensified red stain. (**C**) FLE-treated (30 mg/kg), and (**D**) co-treated with CCl_4_ and FLE (30 mg/kg). (**E**) DLE-treated (30 mg/kg) showing normal collagen deposition and (**F**) co-treated with CCl_4_ and DLE (30 mg/kg).

Sirius Red and Masson’s trichrome staining of hepatic cells are shown in Figure 2 and Figure 3.

Microscopic observations of the hepatic tissues of control group (Figure 2A) and those of groups pretreated with FLE and DLE (Figure 2 and Figure 3C,E) show a normal distribution of collagen fibers between the portal spaces and the centrilobular vein manifested by a low red and blue color. After treatment with CCl_4_ (Figure 2 and Figure 3B), fibrous collagen bridges (shown by the intense red and blue stains) were formed between portal spaces and the centrilobular, marking hepatic fibrosis.

Concerning the association with FLE and DLE, the study revealed that these extracts exerted a protective effect against CCl_4_-induced hepatic fibrotic scarring, observed through the decreasing red and blue staining when compared to that of control (Figure 2 and Figure 3D,F).

### 3.4. Effects of Olive Leaf Extracts on the DNA Fragmentation

**Figure 4:**
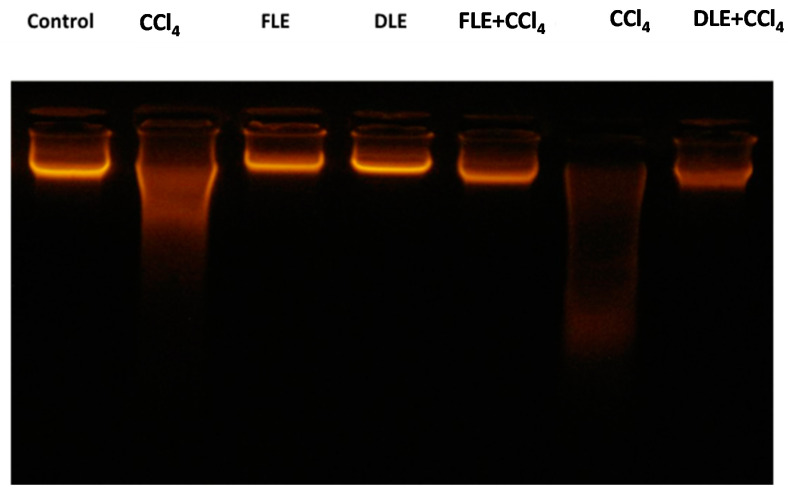
Agarose gel electrophoresis of DNA fragmentation. Lane 1: control hepatic tissue DNA; lane 2 and lane 6: CCl_4_ intoxicated liver DNA, lane 3 and lane 4: FLE- or DLE-treated liver DNA; lane 5 and lane 7: FLE + CCl_4_- or DLE + CCl_4_- treated liver DNA.

As shown in Figure 4, the DNA extracted from the hepatic sections has changed qualitatively. The control group DNA (lane 1) and that of FLE or DLE-treated groups (lanes 3 and 4) showed an intact band. However, we observed that DNA degraded after treatment with the CCl_4_ (lane 2 and lane 6), marked by an obvious smearing and laddering which indicates apoptosis. In the FLE + CCl_4_ and DLE+ CCl_4_ rats (lane 5 and lane 7), the olive leaf extract addition shows its effect on preventing DNA damage and provides results close to those of the control rats, as observed in Figure 4.

## 4. Discussion

Despite recent therapeutic advances, many liver diseases remain relentlessly progressive because specific therapies to target the underlying etiologies of the liver diseases are not available. Therefore, the demand for liver transplantation is likely to increase unless more effective therapeutic and anti-fibrotic agents are developed [45].

In this research work, healthy adult male albino rats were used as an experimental model for the evaluation of olive leaf extracts on induced hepatotoxicity. Among the known hepatotoxic agents, CCl_4_ is the best model of oxidative stress-induced liver damage and is commonly used to assay the hepatoprotective capacity of drugs [46].

The obtained results revealed that the oral gavage of SC-CO_2_ olive leaf extracts effectively attenuated the CCl_4_-induced hepatic damage and fibrotic scarring in rats. The hepatotoxin-caused injury was marked through the antioxidant enzyme markers’ determination in the study of hepatic tissues and DNA. The results obtained in our current study are in accordance with those reported in previous works related to the impact of CCl_4_ on the liver [39,46,47,48,49,50,51]. 

CCl_4_ is reported to produce free radicals, which affect the cellular permeability of hepatocytes, subsequently leading to elevated levels of serum biochemical parameters such as ALT, AST and ALP [51]. This marks a damaged structural and functional integrity of the liver cell membranes, since these cytosolic enzymes are only released into circulation after hepatic cellular damage. An increase in ALT enzyme activity is almost always due to hepatocellular damage and is usually accompanied by an increase in AST and ALP. Oral administration of both fresh and dried olive leaf extracts produced a significant decrease in serum ALT, AST and ALP levels in CCl_4_-induced rats, pointing to their hepatoprotective effect. The reduced concentrations of these markers as a result of olive leaf extract administration observed during the present study might be due to the presence of bioactive compounds such as phenolic and triterpenoid compounds; although, there is a difference observed between fresh and dried leaf extracts (Table 1). As in the present study, previous research works on olive leaf extracts showed that CCl_4_ caused significant elevation of ALT, AST, ALP activities, while pretreatment with olive leaf extracts significantly suppresses the increase in their levels [24,25]. It has been reported that effective hepatoprotective agents must suppress the increase in ALT activity and bring it closer to a normal level in order to induce liver-healing. This would suggest that agents that can lower ALP levels may be useful in hepatoprotection [9].

The fluidity of the biological membrane as well as some enzyme activities can be affected by lipid peroxidation [36]. In our research work, we observed how treatment with FLE and DLE extracts significantly reduced lipid peroxidation level in CCl_4_ treated rats, which suggests that the lipid-oxidation inhibition was exerted by the SC-CO_2_ olive leaf extracts. The generated toxic radicals could be masked through the antioxidants present in the extracts. Amongst the reactive species marker of oxidative stress in cells, we find the highly reactive compound MDA [6]. An increase in MDA levels in tissue sections as a result of CCl_4_ treatment signifies that lipid oxidation has increased, which subsequently causes tissue injury and the intrinsic cell system failure to eliminate exogenous hepatotoxin agents. In our study, the treatment with SC-CO_2_ olive leaf extracts showed a significant reduction in lipid peroxidation levels in CCl_4_-treated rats, demonstrating the capacity of the extracts to reduce such severe alterations. The same behavior was observed for the elevated level of protein carbonyl groups considered as an indicator of protein oxidation. Our results are in agreement with those reported in the literature. In fact, when investigating the protective activity of dried olive leaf extract on CCl_4_-induced liver damage in rats, the researchers reported that the concentration of MDA significantly increased in rats administered CCl_4_ when compared to the control group [25]. A similar observation for increasing MDA levels was reported in another study [24] after which a significant decrease was registered in pretreated rats with (methanolic or butanolic) olive leaf extracts in comparison to CCl_4_-treated rats. Moreover, ethanolic olive leaf extract produced a significant decrease in lipid peroxidation levels in the liver of rats with fluoxetine-induced hepatotoxicity [30]. 

GPx is an enzyme with peroxidase activity which assures the protection from oxidative damage [9]. GPx catalyzes the reduction in H_2_O_2_ or organic peroxide to water or alcohol [52]. Other important antioxidant enzymes are SOD and CAT. SOD is a key enzyme with the ability to convert superoxide radicals into hydrogen peroxide and molecular oxygen, while CAT enhances the conversion of H_2_O_2_ to water and molecular oxygen.

In this study, high activities of GPx, SOD and CAT were observed following CCl_4_ intoxication. Similar observation for SOD was reported in another research on the hepatoprotective effect of extracts of *Cnestis ferruginea* against CCl_4_-induced toxicity [46]. High antioxidant enzyme activities of tissues and organs do not mean efficient protective potential against oxidative stress caused by CCl_4_ intoxication. It was found that antioxidant enzyme mRNA expression and enzyme activity were induced by the exposure of hepatocytes to hydrogen peroxide for 24 h [53]. Thus, the increase in CAT, SOD, and GPx activities observed in the liver are probably partly due to the elevated hydrogen peroxide content generated during the metabolism of CCl_4_ in the body, which lead to up-regulation of gene expression of antioxidant enzyme mRNA and consequently induced an increase in their activities in the liver tissue [54]. The elevated catalase and GPX activities in liver tissue may indicate a large increase in hydrogen peroxide; however, we cannot state to what degree the process of hydrogen peroxide removal is effective [55].

Additionally, the pretreatment of CCl_4_-intoxicated rats with FLE or DLE showed a marked decrease in the activities of antioxidant enzymes compared to the CCl_4_-only group. The protective effect of the extracts in CCl_4_-treated rats is manifested by their capacity to maintain the redox balance marked by the levels of antioxidant enzymes that changed to a figure close to that registered for the control group [9].

Histopathological studies also support the biochemical analysis. The liver histology was improved after co-treatment with SC-CO_2_ extracts in comparison to the CCl_4_ only-treated group, suggesting the hepatoprotective capacity of tested extracts against tissue fibrosis. Olive leaves exerted a significant protective effect against CCl_4_-induced liver damage as marked by the improvement of serum oxidative stress biomarkers and the histology of tissue sections. 

The results also showed that CCl_4_ administration could induce hepatic fibrosis detected by Sirius Red and Masson’s Trichrome staining, as manifested by excessive collagen deposition in the hepatic tissue. These findings were in line with other studies showing that CCl_4_ is able to induce liver fibrosis [56]. The fibrosis status could be related to the activation of hepatic stellate cells (HSCs) as a result of elevated levels of a variety of inflammatory cytokines induced by CCl_4_ intoxication, as reported in previous studies [57].

Administering FLE or DLE along with CCl_4_ significantly reduced liver fibrosis. These data support the efficacy of FLE or DLE in reducing the hepatocellular toxic effects of CCl_4_ and in suppressing the activation and proliferation of HSCs. This positive effect could be due to inflammation reduction. These findings are in line with recent studies that indicate that herbal bioactive molecules are effective against fibrosis in hepatic tissues [58,59].

Despite that significant effect observed for both DLE and FLE extracts, the different composition of tested extracts highlights the importance of phenolic compounds from different classes and triterpenoids as bioactive compounds. Concerning chemical differences related to phenolic compounds, it has to be taken into account that the DLE extract was characterized by the presence of high quantities of hydroxytyrosol and oleuropein isomer. Evaluating the hepatoprotective effect of oleuropein purified from olive leaves, it was found that its administration during ethanol-induced toxicity in rats improves the antioxidant defense system and reduces the levels of lipid peroxides [5]. In another study, oleuropein- and hydroxytyrosol-rich olive leaf extracts revealed hepatoprotective effects against high-fat diet-induced metabolic disorders in rats [14]. With regard to triterpenoids, maslinic acid was only detected and quantified in the FLE extract. Nevertheless, the hepatoprotective effects of triterpenoids present in SC-CO2 olive leaf extracts have not been reported previously. 

Since active phenolics and triterpenoids may have not only additive but also synergic effects on hepatoprotection, future investigations are warranted to develop purified triterpenoid extracts and determine correlations between the olive leaf isolated compounds and the hepatoprotective activity parameters.

## 5. Conclusions

The findings of the present study revealed the capacity of olive leaf extracts obtained by supercritical CO_2_ extraction in repairing injuries caused by CCl_4_-induced hepatotoxicity in a rat model. The obtained results show that fresh and dried olive leaf extracts are able to ameliorate the perturbed biochemical parameters caused by CCl_4_ treatment. Moreover, SC-CO_2_ olive leaf extract administration resulted in a reduction in elevated levels of liver lipid peroxidation, and a reduction in protein carbonyls due to CCl_4_ administration was observed. Serum biochemical markers affirmed the capacity of SC-CO_2_ extracts to protect DNA, and histopathological studies confirmed the capacity of olive leaf extracts to improve liver fibrosis caused by CCl_4_ treatment. Therefore, olive leaf contributes to health benefits and it is a potential source of powerful antioxidant compounds. In conclusion, the current research indicates that SC-CO_2_ extracts from olive leaves have an in vivo protective effect against CCl_4_-induced injury in rats’ livers. Such a biological study is extremely important to highlight the usefulness of olive tree leaves among plants stated as having hepatoprotective activities. Olive leaves, precious by-products from which we have recovered bioactive compounds by green extraction technology, can be reused for agronomic, food, nutraceutical, and pharmaceutical applications.

## Figures and Tables

**Table 1:** Identified and quantified phytochemicals in the SC-CO_2_ olive leaf extracts.

*m*/*z* [M − H]^−^	Molecular Formula	Compound	FLE	DLE	Classification
389.1089	C_16_H_22_O_11_	Secologanoside	-	0.15 ± 0.2	Phenolic compound
153.0557	C_8_H_10_O_3_	Hydroxytyrosol	-	19 ± 1	Phenolic compound
403.1246	C_17_H_24_O_11_	Elenolic acid glucoside isomer 1	-	0.05 ± 0.01	Phenolic compound
151.0401	C_8_H_8_O_3_	Vanillin	3 ± 0.3	13.0 ± 0.4	Phenolic compound
403.1246	C_17_H_24_O_11_	Elenolic acid glucoside isomer 2	-	0.70 ± 0.01	Phenolic compound
193.0506	C_10_H_10_O_4_	Ferulic acid	-	0.40 ± 0.01	Phenolic compound
539.177	C_25_H_32_O_13_	Oleuropein isomer 1	-	42 ± 1	Phenolic compound
539.177	C_25_H_32_O_13_	Oleuropein isomer 2	-	1.10 ± 0.01	Phenolic compound
417.1555	C_22_H_26_O_8_	Syringaresinol	2.4 ± 0.1		Phenolic compound
357.1344	C_20_H_22_O_6_	Pinoresinol	1.1 ± 0.3	0.8 ± 0.2	Phenolic compound
415.1398	C_22_H_24_O_8_	Acetoxypinoresinol	37 ± 1	1 ± 0.1	Phenolic compound
299.0561	C_16_H_12_O_6_	Diosmetin	2 ± 0.2	0.040 ± 0.002	Phenolic compound
471.348	C_30_H_48_O_4_	Maslinic acid	935 ± 7	-	Triterpenoid
455.3531	C_30_H_48_O_3_	Oleanolic acid	1902 ± 8	867 ± 6	Triterpenoid
455.3531	C_30_H_48_O_3_	Ursolic acid	1539 ± 11	1479 ± 7	Triterpenoid
401.1453	C_18_H_26_O_10_	Benzyl alcohol pentose	-	0.05 ± 0.00	Other polar compound

Value = X ± SD (µg of compound per 30 mg of extract), not detected (-).

**Table 2:** Effect of CCl_4_, FLE and DLE administration on hepatic biochemical markers.

	AST (U/L)	ALT (U/L)	ALP (U/L)	LDH (U/L)
**Control**	355 ± 8	285 ± 4	161 ± 5	205 ± 5
**CCl_4_**	417± 4 ***	336 ± 9 ***	231.9± 0.8 ***	261 ± 5 ***
**FLE**	371 ± 4	276 ± 2	171 ± 4	199 ± 8
**CCl_4_ + FLE**	402 ± 3 ^+^	308 ± 9 ^++^	207 ± 2 ^++^	240 ± 5 ^++^
**DLE**	354 ± 5	281 ± 6	158 ± 9	207 ± 7
**CCl_4_ + DLE**	389 ± 4 ^+++^	316 ± 3^+^	211 ± 3 ^+^	242 ± 4 ^+^

AST: aspartate aminotransferase; ALT: alanine aminotransferase; ALP: alkaline, phosphatase and LDH: lactate dehydrogenase. Values are expressed as mean ± SD of six rats in each group. CCl_4_ group versus control group: ** *p* < 0.01; *** *p* < 0.001. CCl_4_ + FLE or CCl_4_ + DLE group versus CCl_4_ group: ^+^ *p* < 0.05; ^++^ *p* < 0.01; ^+++^ *p* < 0.001.

**Table 3:** Effect of CCl_4,_ FLE and DLE on liver oxidative stress.

	TBARS (nmol MDA Equivalents/g Tissue)	PC (nmol/mg Protein)	SOD (U/mg Protein)	CAT (µmol of H_2_O_2_ Destroyed/min per mg Protein)	GPx (nmol of NADPH Oxidized/min per mg Protein)
**Control**	1.13 ± 0.2	1.0 ± 0.1	89 ± 3	109 ± 8	83 ± 3
**CCl_4_**	4.6 ± 0.3 ***	2.7 ± 0.3 ***	141 ± 9 ***	136 ± 6 **	100 ± 4 **
**FLE**	1.4 ± 0.4	0.9± 0.2	78 ± 4	98 ± 4	73 ± 4
**CCl_4_ + FLE**	2.9 ± 0.7 ^+^	1.6 ± 0.1 ^+++^	125 ± 3 ^+^	119 ± 4 ^+^	81 ± 1 ^++^
**DLE**	1.0 ± 0.7	1.1 ± 0.2	87 ± 2	98 ± 9	74 ± 7
**CCl_4_ + DLE**	2.7 ± 0.4^++^	2.03 ± 0.05 ^++^	123 ± 5 ^++^	120 ± 1 ^+^	87 ± 5 ^+^

Values are expressed as mean ± SD of six rats in each group. CCl_4_ group versus control group: ** *p* < 0.01; *** *p* < 0.001. CCl_4_ + FLE or CCl_4_ + DLE group versus CCl_4_ group: ^+^ *p* < 0.05; ^++^ *p* < 0.01; ^+++^ *p* < 0.001.
